# High-Speed Fluorescence Imaging and Intensity Profiling of Femtosecond-Induced Calcium Transients

**DOI:** 10.1155/IJBI/2006/93438

**Published:** 2006-03-07

**Authors:** Daniel Day, Charles G. Cranfield, Min Gu

**Affiliations:** Centre for Micro-Photonics, Faculty of Engineering and Industrial Sciences, Swinburne University of Technology, P.O. Box 218, Hawthorn VIC 3122, Australia

## Abstract

We have demonstrated a combined imaging system, where the physiology of biological specimens can be imaged and profiled at 10–20 frames per second whilst undergoing femtosecond laser irradiation. Individual GH3 cells labeled with the calcium fluorophore Fluo-3 were stimulated using a counter-propagating focused femtosecond beam with respect to the imaging system. As a result of the stimulation, calcium waves can be generated in COS cells, and laser-induced calcium oscillations are initiated in the GH3 cells. Single-photon fluorescence images and intensity profiles of the targeted specimens are sampled in real-time using a modified PerkinElmer UltraView LCI microscope.


Hindawi Publishing Corporation
International Journal of Biomedical Imaging
Volume 2006, Article ID 93438, Pages 1–6
DOI 10.1155/IJBI/2006/93438

### High-Speed Fluorescence Imaging and Intensity Profiling of Femtosecond-Induced Calcium Transients

**Daniel Day, Charles G. Cranfield, and Min Gu**

*Centre for Micro-Photonics, Faculty of Engineering and Industrial Sciences, Swinburne University of Technology, P.O. Box 218, Hawthorn VIC 3122, Australia*

Received 21 September 2005; Revised 14 December 2005; Accepted 5 January 2006

Recommended for Publication by Vasilis Ntziachristos

We have demonstrated a combined imaging system, where the physiology of biological specimens can be imaged and profiled at 10–20 frames per second whilst undergoing femtosecond laser irradiation. Individual GH3 cells labeled with the calcium fluorophore Fluo-3 were stimulated using a counter-propagating focused femtosecond beam with respect to the imaging system. As a result of the stimulation, calcium waves can be generated in COS cells, and laser-induced calcium oscillations are initiated in the GH3 cells. Single-photon fluorescence images and intensity profiles of the targeted specimens are sampled in real-time using a modified PerkinElmer UltraView LCI microscope.

Copyright © 2006 Daniel Day et al. This is an open access article distributed under the Creative Commons Attribution License, which permits unrestricted use, distribution, and reproduction in any medium, provided the original work is properly cited.

### 1. INTRODUCTION

Continued development of optical systems for simultaneous observation and manipulation of live biological specimens has produced advances in understanding cell physiology. Traditional optical microscopes have given way to multifunctional, multilaser-based observation platforms that provide us with the opportunity to interact with the specimen on a subcellular level.

Fluorescence signals of cells can be linked to the overall health and integrity of those cells [1], with fluctuations in the signals indicating effects such as changes in dye loading, fluorescence resonance energy transfer (FRET), fluorescence lifetime imaging (FLIM), fluorescence recovery after photobleaching (FRAP), fluorescence loss in photobleaching (FLIP), cell activation, and cell destruction. Monitoring the integrity of biological specimens that are being altered due to focused femtosecond (fs) irradiation is important to ensure that no damage is being caused by such illumination. While limited exposure to high peak intensity laser pulses has been demonstrated as a successful tool in microsurgical applications [2–15] and to a lesser extent cell trapping [16, 17], continued monitoring of cell vitality during exposure and post-exposure will help to explain the intracellular and extracellular processes that occur as a result of the exposure.

There are numerous advantages to using femtosecond lasers in the near infrared (NIR) for manipulating cells as compared with typical single-photon-induced photo effects, such as highly localized nonlinear photodamage, increased penetration depth, and limited heat transfer to sample. The finite interaction period of a femtosecond pulse with a cell provides a mechanism for altering particular cell characteristics without leading to the destruction of the cell. Recently femtosecond lasers have been demonstrated as a method for noninvasive procedures like dissection [15], photodisruption [12, 18, 19], microinjection [13, 14], and cell transfection [3]. The use of femtosecond irradiation has also been used to generate a calcium ion response in HeLa cells [18] and neocortical neurons in rat brain slices [19]. Imaging samples in real time (typically 25–30 frames per second) have a couple of advantages over standard beam scanning based modalities. The Nipkow disk [20] reduces the high levels of photobleaching associated with beam scanning configurations whilst allowing millisecond (ms) monitoring of fluorescence intensity and/or spectrum across the desired samples.

In this paper, we demonstrate the use of real-time single-photon fluorescence monitoring of cells that are activated using a femtosecond laser beam. Compared with standard commercial fluorescence microscopes that use a beam scanning method coupled with photomultiplier tubes (PMTs),

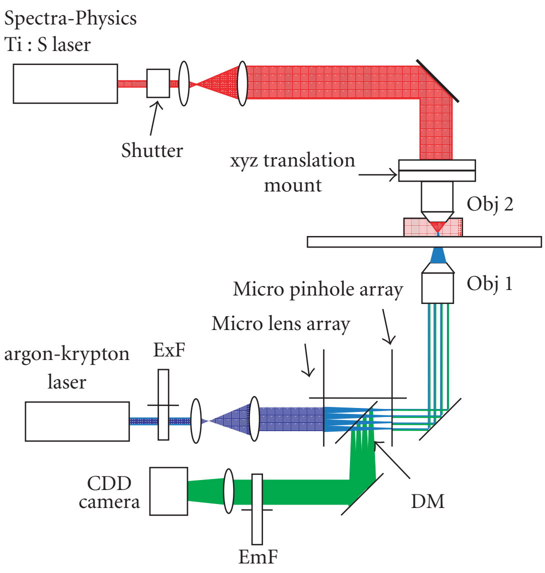

Figure 1: Schematic diagram of the modified PerkinElmer real-time scanning system with counter-propagating focused femtosecond irradiation.

the time resolution of the real-time system allows detection of cellular response to femtosecond irradiation in previously undetectable time periods. Traditional beam scanning microscopes can take up to one second to produce an image, which could lead to distorted images of relatively high-speed cellular activity. A Nipkow disk scanning microscope can produce up to hundreds of frames a second depending on the rotational speed of the Nipkow disk and the data transfer rate of the CCD camera. The fast imaging times used allow this system to monitor the speed by which calcium waves traverse a cell following a short femtosecond laser pulse. Here we also provide examples of calcium ion ($Ca^{2+}$) oscillations being instigated as a result of femtosecond laser pulses.

### 2. EXPERIMENTAL SETUP

The schematic diagram of the modified PerkinElmer Ultraview LCI microscope is illustrated in Figure 1. The PerkinElmer real-time scanning unit is connected to an Olympus IX71 inverted microscope. The excitation source utilized in the real-time system is a fiber coupled argon-krypton laser with excitation bandpass filters at the wavelengths 488 nm, 547 nm, and 647 nm on a stepper motor controlled filter wheel (ExF). The excitation beam is then passed through two matched, rotating disks. The first disk contains an array of micro lenses which focus the light through the array of pinholes on the second disk (Nipkow disk). From there the light enters the IX71 microscope and is focused on the sample with an Olympus LUCPlanFL 40 × 0.6 objective (Obj 1). The fluorescence light is collected with the same objective and passed back through the rotating pinhole array where the dichroic mirror (DM) reflects the fluorescence into the charge coupled detector (CCD). Another stepper motor controlled filter wheel (EmF) located before the CCD contains the emission filters.

The source for the femtosecond beam is a Spectra-Physics MaiTai titanium:sapphire femtosecond pulsed laser which produces 80 fs pulses at a repetition rate of 80 MHz and an average power of 950 mW. The MaiTai has a tunable wavelength range from 730 nm to 870 nm, which allows multiphoton excitation of most biological dyes and specimens. The pulse width of the MaiTai laser is not precompensated before being focused on the sample. It is expected that due to group velocity dispersion (GVD), there is a broadening of the femtosecond pulse as it passes through the expansion optics and objective lens (Obj 2). Previous research on measuring the temporal broadening of a femtosecond pulse through high numerical aperture objective lenses [21, 22] indicates that there is approximately a 50% increase in the pulse width. Further research has also demonstrated that temporal broadening through 1 m of single-mode optical fibre only produces a 3.2 fs pulse from the original 80 fs pulse [23]. It is therefore projected that the pulse width used in these experiments is approximately 200 fs by the time the pulse is focused on the sample.

The femtosecond beam is passed through a mechanical shutter that is used to control the exposure time from milliseconds to hours. The beam is then expanded and directed through the modified transmission head of the IX71 microscope, where it is directed down through an Olympus LUMPlanFL/IR 60 × 0.9 W objective (Obj 2) into the sample. An over expanded top-hat beam profile is used so that when the objective is translated, there is no change in the profile of the focus spot. The objective is mounted in a computer-controlled $x$-$y$ stepper motor scanning stage which is used to control the focus spot within the field of view of the imaging objective. A piezo scanner attached to the objective is used to position the focus spot in the $z$ axis.

The system can also be used to directly image and monitor the two-photon signal coming from the femtosecond focus spot in a cell by stopping the single-photon excitation beam with an appropriate beam block in the excitation filter wheel.

### 3. EXPERIMENTS

Experiments to determine the effectiveness of the modified system for cell activation using a counter-propagating femtosecond laser beam, with respect to the imaging system, were conducted on two types of cells. GH3 rat pituitary cells and COS cells, a simian fibroblast cell line, were used. Both cell types were grown in 35 mm diameter culture dishes with a 170 $\mu$m coverglass bottom (MatTek Corp.). The cells were cultured in Dubelco's modified eagle medium (DMEM) with 20 mM HEPES, 10% fetal bovine serum, 2% penicillin-streptomycin, and 200 mM L-glutamine solution (Sigma Aldrich) in a $CO_2$ incubator at 37°C. The cells were then loaded with 2 $\mu$M Fluo-3 AM (Molecular Probes) for 30 minutes at room temperature in DMEM without supplements. After loading, the cells were then washed and finally immersed in 2 mL of DMEM solution with 20 mM HEPES again without the supplements.

### *3.1. Femtosecond laser-induced calcium oscillations*

Femtosecond laser irradiation of GH3 cells can be used to induce $Ca^{2+}$ oscillations. The oscillations in the targeted cell (cell 1) seen in Figure 2 were initiated in direct response to a 15 millisecond exposure of an 800 nm, 80 fs beam.

The average power of the laser in the focus spot is 8 mW. To image the cells, they were excited with the 488 nm laser line from the Ar-Kr laser and the corresponding fluorescence was collected after it passed through the 525 nm filter. The cells were imaged for 54 seconds before cell (1) was exposed to the femtosecond laser beam and then imaged a further 146 seconds to monitor the oscillations. For such an extended imaging period, the frequency of image capturing was decreased to 2 frames per second to reduce potential photobleaching. The oscillating fluorescence intensity levels measured in Figures 2(b)–2(e) were an average of the fluorescence intensity over the whole cell.

The oscillations generated in cell (1) as a result of the laser stimulus triggered oscillations in the neighboring cells (2), (3), and (4) in response to the possible release of chemical factors. The exact chemical mechanism that triggers the oscillations in neighboring cells is not fully understood but it is possibly due to the release of extracellular chemical factors [24] (e.g., growth hormone). The exposure to the femtosecond laser beam results in no permanent damage to the cells, including the targeted cells, during the period of monitoring as evident by the continued fluctuations in $Ca^{2+}$ concentrations.

### *3.2. Femtosecond laser-induced calcium wave*

The same process used to induce oscillations in GH3 cells can be used to increase the $Ca^{2+}$ levels in COS cells. The focused femtosecond laser beam induces a localized increase in

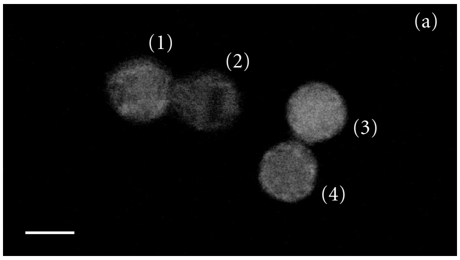

(a)

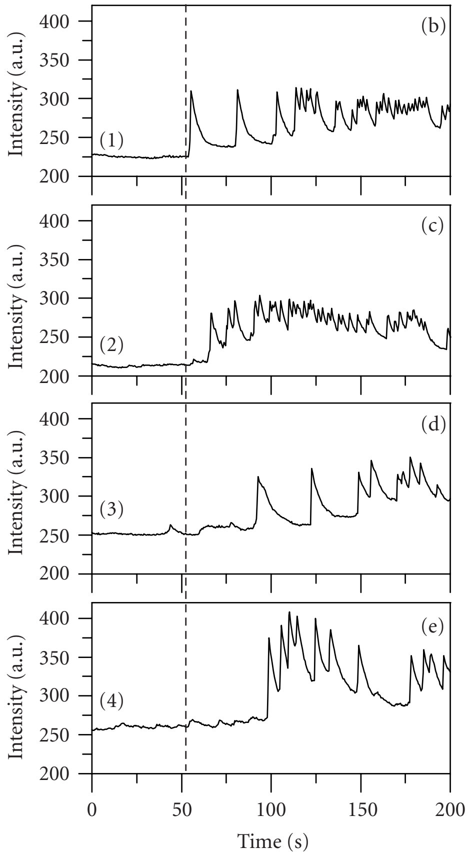

(b)

FIGURE 2: Fluorescence intensity profiles of femtosecond laser-induced calcium oscillations in GH3 cells versus time. (a) Single-photon fluorescence image of GH3 cells. (b), (c), (d), and (e) intensity profiles of cells (1), (2), (3), and (4), respectively. Cell (1) was exposed to the femtosecond laser at the time indicated by the dashed line. The scale bar is 10 $\mu$m.

the fluorescence signal which is followed by a $Ca^{2+}$ wave that travels accross the length of the cell, increasing the overall cell fluorescence with it. The $Ca^{2+}$ wave expands radially away from the point of interaction, slowing gradually the further it travels. Figure 3 shows the increase in fluorescence signal over time.

Due to the high frame rate of the imaging system, it is possible to measure the velocity of the $Ca^{2+}$ wave as it expands across the cell. Figure 4 shows the decrease in the

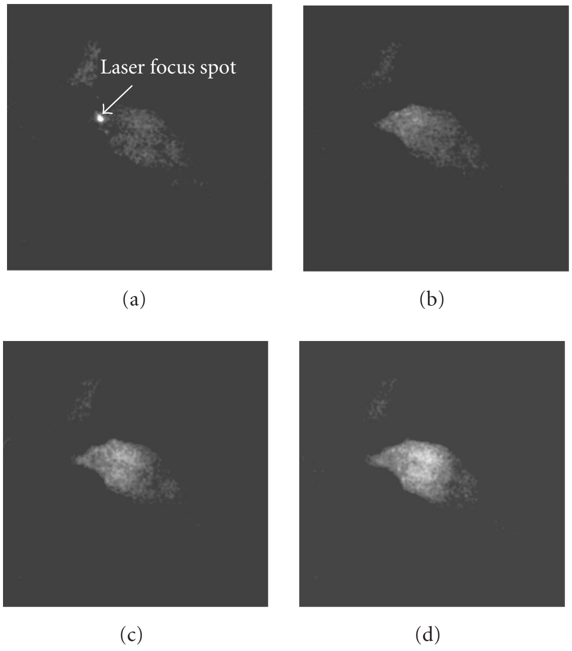

Figure 3: Femtosecond laser induced $Ca^{2+}$ wave expanding across a COS cell. Selected images represent images taken at (a) 0 second, (b) 0.4 seconds, (c) 0.8 seconds, and (d) 1.4 seconds after exposure. The scale bar is 10 $\mu$m.

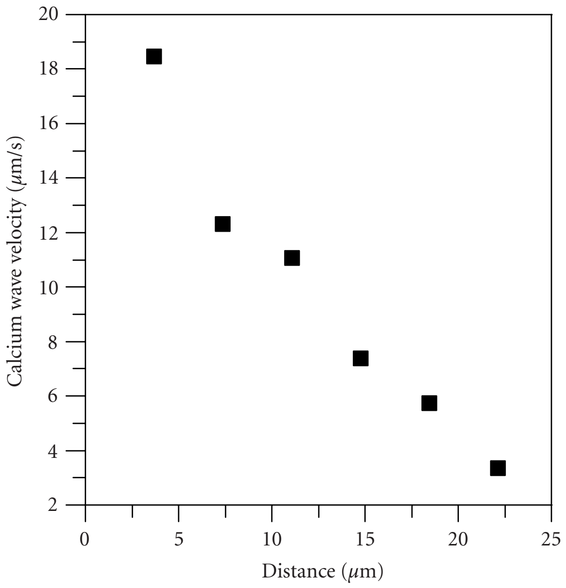

Figure 4: Velocity profile of a $Ca^{2+}$ wave as it expands across a COS cell.

measured velocity of the $Ca^{2+}$ wave as it travels along the distance of the cell. The cell was exposed to the femtosecond irradiation for 25 milliseconds with an average power of 15 mW in the focus. The capture rate of the images was increased to 10 frames per second in order to image the traveling $Ca^{2+}$ wave. The limitation on the frame rate is the strength of the fluorescence signal of the cell. A weaker fluorescence signal requires a longer exposure time and therefore a slower image capture rate.

### 4. DISCUSSION

The combined use of a real-time imaging system and targeted femtosecond irradiation can now be used to study various aspects of cell physiology on a time scale not previously achieved. The use of the counter-propagating femtosecond beam provides the freedom to manipulate the sample in three dimensions while maintaining a simplified imaging system.

We have also demonstrated that femtosecond pulsed lasers can be used to initiate intracellular $Ca^{2+}$ oscillations, and that this effect might also be useful in initiating oscillations in adjacent cells. We postulate that this trigger of oscillations in neighboring cells is due to extracellular chemical factors being released from the GH3 cell as a result of the femtosecond pulse. The release of these chemical factors then affect the neighboring cells, inducing comparable oscillations in them. We have noticed that cells do not have to be touching for this to occur. Caution should be used in interpreting these results, however, as GH3 cells can show spontaneous oscillations. This ability to induce oscillations in adjacent cells, when used in conjunction with various chemical antagonists, could open the way for novel methods of micropharmacological research.

The rapid propagation of a calcium wave across a cell often requires real-time imaging in order to observe it fully. Our system can follow this propagation from the instant the cell was irradiated with the femtosecond laser.

This system's ability to micro manipulate the counterpropagating objective for the femtosecond pulsed laser allows for the cells to be femtosecond trapped, and moved. The added ability to monitor cells in real-time means that the vitality of the cells can be monitored simultaneously.

### ACKNOWLEDGMENT

The authors would like to thank the Australian Research Council for its support.

### REFERENCES

[1] J. White and E. H. Stelzer, "Photobleaching GFP reveals protein dynamics inside live cells," *Trends in Cell Biology*, vol. 9, no. 2, pp. 61–65, 1999.

[2] U. K. Tirlapur and K. König, "Technical advance: near-infrared femtosecond laser pulses as a novel non-invasive means for dye-permeation and 3D imaging of localised dye-coupling in the Arabidopsis root meristem," *The Plant Journal*, vol. 20, no. 3, pp. 363–370, 1999.

[3] U. K. Tirlapur and K. König, "Cell biology: targeted transfection by femtosecond laser," *Nature*, vol. 418, no. 6895, pp. 290–291, 2002.

[4] H. Oehring, I. Riemann, P. Fischer, K.-J. Halbhuber, and K. König, "Ultrastructure and reproduction behaviour of single

CHO-K1 cells exposed to near infrared femtosecond laser pulses," *Scanning*, vol. 22, no. 4, pp. 263–270, 2000.

[5] U. K. Tirlapur and K. König, "Femtosecond near-infrared laser pulses as a versatile non-invasive tool for intra-tissue nanoprocessing in plants without compromising viability," *The Plant Journal*, vol. 31, no. 3, pp. 365–374, 2002.

[6] K. König, I. Riemann, P. Fischer, and K.-J. Halbhuber, "Intracellular nanosurgery with near infrared femtosecond laser pulses," *Cellular and Molecular Biology*, vol. 45, no. 2, pp. 195–201, 1999.

[7] K. König, P. T. C. So, W. W. Mantulin, B. J. Tromberg, and E. Gratton, "Cellular response to near-infrared femtosecond laser pulses in two-photon microscopes," *Optics Letters*, vol. 22, no. 2, pp. 135–136, 1997.

[8] K. König, T. W. Becker, P. Fischer, I. Riemann, and K.-J. Halbhuber, "Pulse-length dependence of cellular response to intense near-infrared laser pulses in multiphoton microscopes," *Optics Letters*, vol. 24, no. 2, pp. 113–115, 1999.

[9] M. W. Berns, J. Aist, J. Edwards, et al., "Laser microsurgery in cell and developmental biology," *Science*, vol. 213, no. 4507, pp. 505–513, 1981.

[10] N. Shen, M. Colvin, F. Genin, et al., "Using femtosecond laser subcellular surgery to study cell biology," *Biophysical Journal*, vol. 86, part 2, supplment S, pp. 520A–520A, 2004.

[11] B. C. Stuart, M. D. Feit, S. Herman, A. M. Rubenchik, B. W. Shore, and M. D. Perry, "Optical ablation by high-power short-pulse lasers," *Journal of the Optical Society of America B: Optical Physics*, vol. 13, no. 2, pp. 459–468, 1996.

[12] W. Watanabe, N. Arakawa, S. Matsunaga, et al., "Femtosecond laser disruption of subcellular organelles in a living cell," *Optics Express*, vol. 12, no. 18, pp. 4203–4213, 2004.

[13] S. K. Mohanty, M. Sharma, and P. K. Gupta, "Laser-assisted microinjection into targeted animal cells," *Biotechnology Letters*, vol. 25, no. 11, pp. 895–899, 2003.

[14] W. Tao, J. Wilkinson, E. J. Stanbridge, and M. W. Berns, "Direct gene transfer into human cultured cells facilitated by laser micropuncture of the cell membrane," *Proceedings of the National Academy of Sciences of the United States of America*, vol. 84, no. 12, pp. 4180–4184, 1987.

[15] V. Venugopalan, A. Guerra III, K. Nahen, and A. Vogel, "Role of laser-induced plasma formation in pulsed cellular microsurgery and micromanipulation," *Physical Review Letters*, vol. 88, no. 7, p. 078103, 2002.

[16] K. König, "Laser tweezers are sources of two-photon excitation," *Cellular and Molecular Biology*, vol. 44, no. 5, pp. 721–733, 1998.

[17] B. Agate, C. T. A. Brown, W. Sibbett, and K. Dholakia, "Femtosecond optical tweezers for in-situ control of two-photon fluorescence," *Optics Express*, vol. 12, no. 13, pp. 3011–3017, 2004.

[18] N. I. Smith, K. Fujita, T. Kaneko, et al., "Generation of calcium waves in living cells by pulsed-laser-induced photodisruption," *Applied Physics Letters*, vol. 79, no. 8, pp. 1208–1210, 2001.

[19] H. J. Koester, D. Baur, R. Uhl, and S. W. Hell, "$Ca^{2+}$ fluorescence imaging with pico- and femtosecond two-photon excitation: signal and photodamage," *Biophysical Journal*, vol. 77, no. 4, pp. 2226–2236, 1999.

[20] A. Ichihara, T. Tanaami, K. Isozaki, et al., "High-speed confocal fluorescence microscopy using a Nipkow scanner with microlenses for 3D-imaging of single fluorescent molecule in real time," *Bioimages*, vol. 4, no. 1, pp. 52–62, 1996.

[21] P. E. Hänninen and S. W. Hell, "Femtosecond pulse broadening in the focal region of a two-photon fluorescence microscope," *Bioimaging*, vol. 2, no. 3, pp. 117–121, 1994.

[22] M. Müller, J. Squier, and G. J. Brakenhoff, "Measurement of femtosecond pulses in the focal point of a high-numerical-aperture lens by two-photon absorption," *Optics Letters*, vol. 20, no. 9, pp. 1038–1040, 1995.

[23] D. Bird and M. Gu, "Two-photon fluorescence endoscopy with a micro-optic scanning head," *Optics Letters*, vol. 28, no. 17, pp. 1552–1554, 2003.

[24] A. H. Tashjian Jr., Y. Yasumura, L. Levine, G. H. Sato, and M. L. Parker, "Establishment of clonal strains of rat pituitary tumor cells that secrete growth hormone," *Endocrinology*, vol. 82, no. 2, pp. 342–352, 1968.

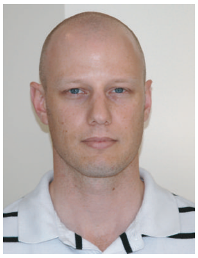

**Daniel Day** received his B.S. degree from Griffith University, Brisbane, Australia in 1996 with majors in physics and mathematics, and in 1997, received First Class Honours from Victoria University of Technology, Melbourne, Australia in optics. He gained his Ph.D. from Swinburne University of Technology, Melbourne, Australia in 2001, researching three-dimensional optical data storage. His Ph.D. thesis was "Three-Dimensional Bit Optical Data Storage in a Photorefractive Polymer." Recently, he completed a Graduate Certificate in Entrepreneurship and Innovation at Swinburne University of Technology. In 1998, he received the Australian Optical Society Postgraduate Student Award for his research into three-dimensional optical data storage. He is a Member of the Australian Institute of Physics, Australian Optical Society, and the Optical Society of America. Since completing his Ph.D., he has established the company 3DCD Technology Pty. Ltd. with Swinburne University which now holds several international patents in three-dimensional optical data storage. Currently, he is a Research Fellow in the Centre for Micro-Photonics at Swinburne University of Technology, Melbourne, Australia, conducting research into three-dimensional microfluidics and fabrication of three-dimensional micro-environments for biological applications.

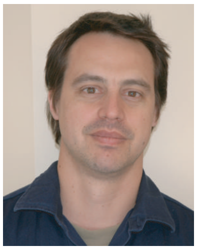

**Charles G. Cranfield** was born in Melbourne, Australia, in 1971. He received the B.S. degree with honours in pharmacology from Monash University, Melbourne, Australia, in 1996. This was followed by the Ph.D. in biophysics from Swinburne University of Technology, Melbourne, Australia, in 2002. His research was about possible mobile telephone RF effects on calcium ion levels in human T lymphocytes. He was invited to discuss these findings to researchers at the Babraham Institute, Cambridge, UK. He then completed 2 years as a postdoctoral researcher in Proffessor Jon Dobson's Nano-Magnetics Laboratory, Keele University, UK. His work there culminated in the discovery of biogenic magnetite in the nematode *C. elegans*. This research was the basis for an article in "Chemistry World" magazine in 2004 entitled "*As the Magnetic Worm Turns; Magnetite Reignites Mobile Phone Radiation Concerns*." At this time, he was also invited to the UK Nanomagnetics Network Meeting to discuss his research. In 2004, he returned to Australia to take up a brief position at Prince Henry's Institute for Medical Research, Melbourne, investigating calcium responses in pituitary cells, before being offered his current position as a Research Fellow of Biophotonics at the Centre for Micro-Photonics, Swinburne University of Technology, Melbourne, Australia.

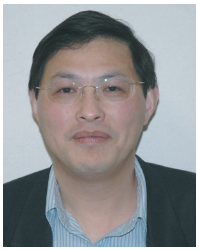

**Min Gu** gained a Ph.D. in optics from the Chinese Academy of Sciences in 1988. He was invited for the appointment of Professor (Chair) of Optoelectronics and Director of the Centre for Micro-Photonics at Swinburne University of Technology from 2000. In 2003, he became a Node Director of the Australian Research Council Centre of Excellence for Ultrahigh-bandwidth Devices for Optical Systems. He is a sole author of two standard reference books, Principles of Three-Dimensional Imaging in Confocal Microscopes and Advanced Optical Imaging Theory. He published over 300 papers in photonic crystals, nanophotonics, micro/nanofabrication, confocal microscopy, laser tweezers, optoelectronic imaging through tissue-like turbid media, laser trapping microscopy, and three-dimensional optical data storage. He is a topical Editor of 2 international optics journals, a Member of the editorial board of another 4 international journals, and was the Guest Editor of Applied Optics and the International Journal of Optical Memory and Neural Networks. He is President and Regional Council Member of the International Society of Optics within Life Sciences. He is also a Fellow of the Australian Institute of Physics, a Fellow of the Optical Society of America, and a Fellow of the International Society for Optical Engineering.

